# Bacteriological diagnosis of β-hemolytic streptococci of the upper respiratory tract

**DOI:** 10.1186/1471-2334-14-S7-P9

**Published:** 2014-10-15

**Authors:** Alina Borcan, Olga Dorobăț, Ioana Bădicuț, Daniela Tălăpan, Mariana Radut, Mona Popoiu, Anca Munteanu, Alexandru Rafila

**Affiliations:** 1 Carol Davila University of Medicine and Pharmacy, Bucharest, Romania; 2 National Institute for Infectious Diseases "Prof. Dr. Matei Balş", Bucharest, Romania; 3 National Institute of Public Health, Bucharest, Romania

## Background

Beta-hemolytic streptococci are major human pathogens associated with local or systemic invasion and post-streptococcal immune disorders. We performed a comparison between bacitracin and latex agglutination test for the identification of beta-hemolytic streptococci. We assessed resistance patterns to erythromycin/clindamycin.

## Methods

Between January 1 2014 – July 15 2014, 5038 throat swabs were sampled from patients hospitalized in INBI Matei Balş according to standard methods. All beta-hemolytic streptococci suspected colonies were tested with bacitracin (0.04%) disks and we used the latex agglutination kit Omega Latex Diagnostic Avipath Strep. Erythromycin resistance testing was performed by disc 2μg Oxoid and clindamycin 2μg Oxoid on the blood M-H medium.

## Results

There were 460 isolates of beta-hemolytic streptococci, bacitracin test was positive for 373 (92.75%). From 87 negative bacitracin test left streptococci, with latex agglutination test, 18 (20.68%) were identified as *Streptococcus pyogenes* (group A) and 69 (79.32%) were group C, G, B, F. *Streptococcus pyogenes* had 8.95% resistance to erythromycin, group C streptococci had 2.12%, and group G 45.45. From strains with resistance to erythromycin 75.6% were with inducible resistance to clindamycin and 17.07% had a constitutive one.

## Conclusion

Latex agglutination is a preferable alternative to bacitracin testing, providing a definite grouping result.

Resistance of *Streptococcus pyogenes* to erythromycin was 8.95% compared with group G which had a resistance of 45.45%.

